# Bone Turnover in Relation to Thyroid-Stimulating Hormone in Hypothyroid Patients on Thyroid Hormone Substitution Therapy

**DOI:** 10.1155/2022/8950546

**Published:** 2022-09-22

**Authors:** Betty Ann Bjerkreim, Sara S Hammerstad, Erik Fink Eriksen

**Affiliations:** ^1^Department of Clinical Endocrinology, Morbid Obesity and Preventive Medicine, Oslo University Hospital, Oslo, Norway; ^2^Institute of Clinical Medicine, Faculty of Medicine, University of Oslo, Oslo, Norway; ^3^Pilestredet Park Specialist Center, Oslo, Norway; ^4^Faculty of Odontology, University of Oslo, Oslo, Norway

## Abstract

**Background:**

Bone turnover markers (BTMs) have emerged as a useful tool for monitoring bone remodeling activity in the skeleton, and their serum levels correlate with bone loss rates in osteoporotic and normal individuals. Whether the same holds for other metabolic bone diseases is still subject to discussion.

**Methods:**

We analyzed the relation between levels of BTMs and TSH in 79 females on thyroid hormone substitution therapy for hypothyroidism. Based on the reference range for TSH (0.2–4.0 mU/L) in our lab, we assessed BTMs in five different groups of patients based on the following criteria: (1) hypothyroidism (TSH >4.0); (2) TSH in the high normal range (1.0–4.0); (3) TSH in the low normal range (0.2–1.0); (4) TSH below the normal range (0.01–0.2); (5) TSH undetectable (<0.01). We studied the relationship between TSH and four different bone markers: procollagen type 1 N-terminal propeptide (PINP), C-terminal cross-linking telopeptide of type 1 collagen (CTX), osteocalcin (OC), and bone specific alkaline phosphatase (BSAP). In a subgroup of patients, bone mineral density was assessed by a DXA scan.

**Results:**

PINP emerged as the most sensitive and dynamic BTM for assessment of bone turnover in this patient group, achieving significant rho values on nonparametric correlation analysis for both TSH (rho −0.47; *p*=0.0001) and FT4 (rho 0.27; *p*=0.018). CTX and OC also revealed significant correlations to TSH, albeit with lower rho values (−0.37 and −0.24, respectively). Categorical analysis showed that bone turnover increased significantly, albeit with pronounced interindividual variability for TSH values below the lower limit of normal (0.2 mU/l), with the most severe affected being women exhibiting suppression of TSH. Further analysis of loss rates by DXA in a limited subgroup of patients showed that this was accompanied by accelerated bone loss.

**Conclusion:**

PINP is the most sensitive marker of bone turnover in thyroid disorders. TSH values below the lower limit of normal are associated with increased bone turnover and accelerated bone loss, however, with pronounced interindividual variations. Assessment of PINP may be a valuable tool in cases where there is concern about possible adverse effects of thyroid hormone substitution therapy on bone.

## 1. Introduction

Thyroid hormones exert powerful effects on bone remodeling. Hyperthyroidism is characterized by increased bone turnover and a negative remodeling balance, causing accelerated bone loss [1–3]. This results in reduced bone mass during active hyperthyroidism, with improvements reported after institution of antithyroid therapy [4, 5]. In most cases, the negative consequences of hyperthyroidism on the skeleton are therefore offset by rapid medical or surgical intervention leading to restoration of euthyroidism and thus normalization of bone turnover [5]. In a large case-control study by Vestergaard et al., an increase in fracture risk was seen within the first 5 years after a diagnosis of hyperthyroidism, but the risk decreased to normal after intervention, be it surgery or antithyroid drugs. Also low dose levothyroxine (LT4) therapy was not associated with increased fracture risk in this study [6]. In an earlier study, the same group reported a reversible decrease in bone mineral density (BMD) associated with increased fracture risk in untreated hyperthyroidism. This risk, however, returned to normal after normalization of the hyperthyroid state [5]. Bone remodeling in hypothyroidism is characterized by reduced turnover and a positive balance between resorption and formation [2, 3, 7], all of which should reduce fracture risk. It is somewhat surprising the large case-control study by Vestergaard et al. found an increase in fracture risk limited to the first 10 years after diagnosis [6]. Whether this is due to the hyperthyroid phase associated with acute thyroiditis remains to be established.

Thyroid hormone over-substitution resulting in TSH suppression is a situation where thyroid hormone excess may persist for years and has been long known to be associated with excessive bone loss and an increased risk of osteoporotic fracture [8, 9]. The exact TSH cut-off where bone loss ensues is, however, still poorly defined. This is a matter of clinical significance because many patients in clinical practice claim to feel better at lower TSH values.

The data on the impact of thyroid hormone suppressive therapy on bone mass and fracture risk is controversial, with some studies claiming no increase in fracture risk [10, 11], while others report significant adverse effects on the skeleton [12, 13]. Data on changes in bone turnover in patients on suppressive thyroid hormone therapy are also controversial. Some studies report increased bone turnover marker (BTM) levels in patients with reduced TSH levels [13, 14] while other studies report no difference [10, 12, 15].

BTMs have emerged as a useful tool for monitoring bone remodeling activity in the skeleton, and their serum levels correlate with bone loss rates [16, 17]. Furthermore, BTMs react more swiftly to changes in remodeling than DXA measurements and are therefore useful for monitoring short-term changes in remodeling activity.

In order to further elucidate the relationship between bone loss and TSH, we therefore investigated levels of BTMs in five different classes of patients on thyroid hormone substitution therapy using the reference range in our lab for TSH (0.2–4.0 mU/L). BTMs were assessed based on the following criteria: (1) hypothyroidism (TSH >4.0); (2) TSH in high normal range (1.0–4.0); (3) TSH in low normal range (0.2–1.0); (4) TSH below normal range (0.01–0.2); and (5) TSH undetectable <0.01.

## 2. Materials and Methods

The study was carried out between 01.02.2020 and 30.06.2022 at Pilestredet Park Specialist Center. A total of 79 patients on thyroid hormone substitution therapy after hypothyroidism due to either thyroiditis or thyroidectomy were included. All had at least two DXA measurements with concomitant assessment of four BTMs (bone specific alkaline phosphatase (BSAP), osteocalcin (OC), procollagen type 1 N-terminal propeptide (PINP), and C-terminal cross-linking telopeptide of type 1 collagen (CTX)). Baseline demographics are shown in Table 1. Exclusion criteria were concomitant medications with known influence on bone metabolism (hormone replacement therapy, bisphosphonates, denosumab, and parathyroid hormone) or concomitant metabolic bone disease.

TSH (reference range: 0.2–4.0 mU/L) was measured with a noncompetitive immunofluorometric analysis by AutoDELFIA (Wallac Oy, Turku, Finland). FT4 (8.0–21.0 pmol/L) was measured with a solid-phase time-delayed fluoro-immunoassay with back-titration by AutoDELFIA (Wallac Oy, Turku, Finland). FT3 (2.8–7.0 pmol/L) was measured with a competitive electrochemiluminescence immunoassay by Cobas e601 (Roche Diagnostics, Indianapolis, IN, USA). CTX (≤0.57 *µ*g/L) and PINP (11–94 *µ*g/L) was measured with a noncompetitive electrochemiluminescence immunoassay by Cobas e601 (Roche Diagnostics, Indianapolis, IN, USA). BSAP (5.5–25.0 *µ*g/L) and OC (1.5–5.4 nmol/L) were measured with a chemiluminescence immunoassay by the Liaison XL kit (DiaSorin Inc., Saluggia, Italy). All analyses were performed at Furst Medical Laboratory, Oslo.

A DXA assessment in a subgroup of patients was performed on a Lunar iDXA (GE Healthcare, Chicago, I. L., USA).

### 2.1. Statistical Analyses

The correlations between continuous variables were analyzed by the Spearman Rank Order Correlation. The differences between categorical TSH values on continuous variables were analyzed by the Kruskal–Wallis Test. All variables were tested for normality by quantile-quantile (QQ)-plots and histograms. Statistical significance was defined as two-tailed *p* < 0.05. All statistical analyses were performed using IBM SPSS Statistics for Windows, version 26 (IBM Corp., Armonk, N. Y., USA).

### 2.2. Ethics

This study was approved by the Regional Ethics Committee for Medical and Health Research (ref. no. 218347).

## 3. Results

### 3.1. Demographics

In Table 1. shows the baseline demographics of patients included in the study. Forty-six patients were treated with LT4 monotherapy, ten with LT4 + Liothyronine (LT3) combination therapy, and four with thyroid extracts (Nature Thyroid (1), Armour (3)). PTH and vitamin D levels did not show significant correlations to TSH. BMD T-scores at the lumbar spine and hip were also unrelated to TSH. Six patients (6.5%) had suffered spine fractures and 24 (28%) had previous peripheral fractures. No correlation between the number of fractures and TSH was demonstrable.

### 3.2. Bone Turnover Marker Levels in Relation to TSH

BTM analyses showed significant correlations between TSH and PINP (*p* < 0.0001), CTX (*p*=0.03), and OC (*p*=0.046), with lower TSH values being associated with higher BTM levels. Comparing the correlation analyses for TSH, FT4, and FT3 versus the four bone markers tested, revealed that PINP emerged as the best marker, achieving the highest rho values and significance values (Table 2).

Categorical analyses revealed that these associations were driven by lower BTM levels in hypothyroid patients (TSH >4.0) and higher levels in patients exhibiting TSH values < 0.01. TSH values in the range 0.2–4.0 showed smaller differences in BTMs. The trends for PINP, CTX, and OC were, however, significant (Kruskal Wallis Test *p*=0.004; *p*=0.03; and *p*=0.046, respectively). OC, however, revealed spurious high values within the normal TSH range in some patients. BSAP did not show any significant trend (*p*=0.535).

**Figure 1:**
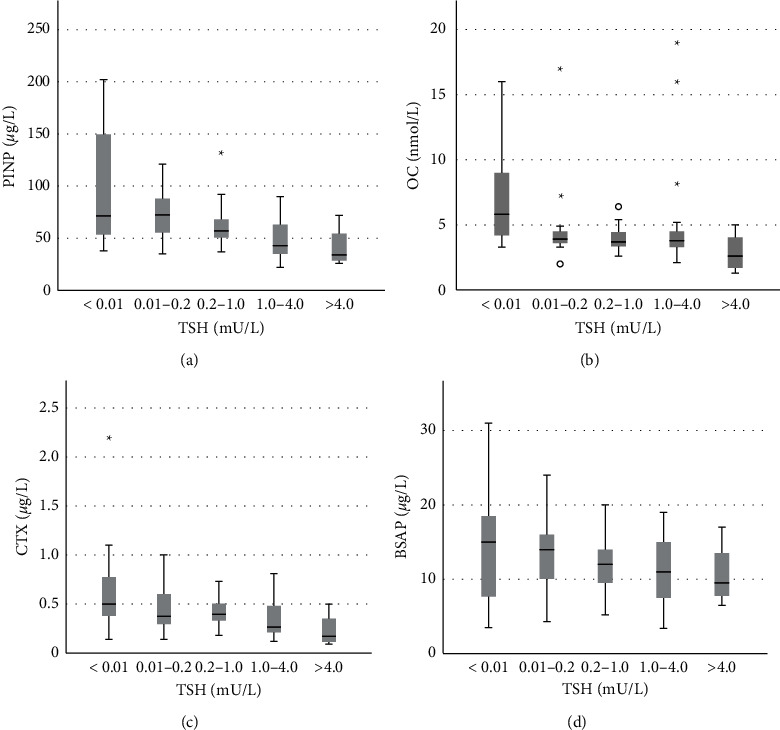
Categorical analysis of the variation in bone turnover markers with TSH. The bone markers investigated were: (a) procollagen type 1 N-terminal propeptide (PINP); (b) osteocalcin (OC); (c) C-terminal cross-linking telopeptide of type 1 collagen (CTX); and (d) bone specific alkaline phosphatase (BSAP).

For TSH levels within the reference range, BTM levels were in the middle of the normal range (Figure 1(a)–1(d)). BTM levels were especially high in patients with undetectable TSH values (although with pronounced variation) (Figure 1(a)–1(d)). A trend towards higher bone turnover levels for patients in the lower half of the normal TSH range vs. the upper half was detectable. While changes in the BTMs CTX, BSAP, and OC were limited within the normal TSH range (0.2–4.0) (Figure 1(b)–1(d)), PINP revealed a steady increase in turnover in that range (Figure 1(a)). PINP levels in the TSH range (0.01–0.2) were in the upper half of the normal range and significantly higher than PINP in those with TSH values between 1.0 and 4.0 (*p* < 0.015) (Figure 1(a)). The interindividual variation was, however, pronounced, and a significant number of patients with undetectable TSH exhibited PINP values in the middle and lower half of the normal range (Figure 1(a)).

When analyzing PINP vs. thyroid function tests (TSH, FT4, and FT3), FT3 exhibited a much larger R^2^-coefficient (*R*^2^ = 0.57) than TSH (*R*^2^ = 0.11) or FT4 (*R*^2^ = 0.12), and may therefore be a better predictor of PINP than TSH and FT4.

### 3.3. Bone Loss in Relation to TSH

**Figure 2:**
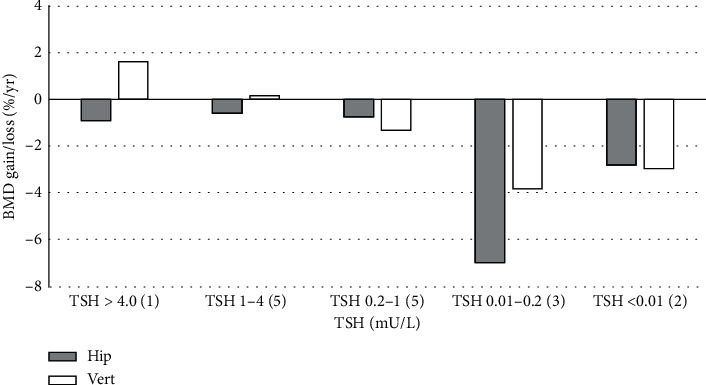
Categorical analysis of the variation in bone mineral density (BMD) loss/gain at the spine (vert) and hip in relation to TSH in a small subgroup of patients where serial BMD measurements were available. TSH values are in mU/L. Number of patients in parentheses.

Figure 2 shows the relationship between TSH values and BMD changes expressed as BMD gain or loss in (%) year. Data were only available in 16 patients. The data show a trend towards excessive bone loss (>2%/year) in patients with TSH below the lower limit of TSH. None of the trends reached significance, however, the trends were consistent for both the hip and spine.

## 4. Discussion

Our BTM analyses corroborate with previous studies demonstrating increased bone turnover in hyperthyroidism and low bone turnover in hypothyroidism as shown by histomorphometry and calcium kinetic studies [7, 18]. Hyperthyroidism results in a pronounced negative bone balance at the level of individual bone remodeling units [1, 19]. Therefore, any increase in bone turnover will further enhance bone loss [1, 19]. In relation to thyroid hormone substitution therapy, our data show the impact of suppressed TSH levels on bone turnover with pronounced interindividual variation. Bone turnover remained within the normal range for a significant proportion of patients. The subgroup analyses revealed that patients with suppressed TSH levels <0.01 experienced significantly increased BTM levels and thus an increased risk of accelerated bone loss. Patients exhibiting TSH values slightly below the normal range (0.01–0.2) revealed increased BTM values compared to patients with TSH values in the upper half of normal. These notions were corroborated by the bone/gain data from a limited number of patients with available DXA. Interindividual variation was profound. However, with a significant number of subjects exhibiting BTM values in the middle of the normal range.

PINP emerged as the most sensitive marker of bone turnover in hypothyroid patients on thyroid hormone substitution therapy, which is in accordance with studies in other metabolic bone diseases [20, 21]. PINP revealed that even slightly suppressed TSH values are associated with increased bone turnover and thus increased bone loss. OC revealed the lowest, albeit significant, Spearman's rho values of the four BTMs. Moreover, some patients exhibited spurious high values within the normal range for TSH, rendering OC the poorest BTM to use in thyroid patients. OC differs from the other markers examined by possessing DNA response elements sensitive to glucocorticoids [22] and vitamin D [23], affecting the osteoblastic synthesis of OC. This may partly explain the larger variation observed compared to the three other BTMs studied.

Previous literature on adverse bone effects of over-substitution is conflicting. Murphy et al. [24] reported a 20–33% increased risk of nonvertebral fractures in patients with elevated FT3 and FT4 levels. Elevated TSH values, on the other hand, were associated with a 35% reduction in risk. This is in line with the findings of Schneider et al. [11], who found reduced volumetric BMD of trabecular bone and the forearm in 28 men and 46 women on suppressive therapy after thyroid cancer surgery.

In a cross-sectional study, Baqi et al. [25] found that women with suppressed TSH values (TSH <0.35 mU/L) did not exhibit higher levels of CTX or PINP. In a smaller cohort of 34 women, Pater et al. reported similar BTM levels when comparing a group of euthyroid women with subjects with TSH <0.35 mU/L. Moreover, no relationship was observed between TSH and bone formation and resorption markers in the whole group of euthyroid postmenopausal women [26].

In their 2006 paper, Lee et al. reported reduced femoral neck BMD in 413 women with subclinical hyperthyroidism. BMD of the spine was similar to controls as were BTMs [27]. In a later paper from 2010, however, they were unable to demonstrate negative skeletal consequences of suppressive thyroid hormone therapy in 94 women after surgery for thyroid cancer [15].

Tsourdi et al. reported significant positive associations between TSH concentrations and PINP, BSAP, OC, and CTX in women but not in men. They also reported that TSH correlated positively with the FRAX score both over the whole TSH range (*p* < 0.01) and within the reference TSH range (*p* < 0.01) [28].

Zofkova and Hill performed a cross-sectional study of 60 euthyroid postmenopausal women. They reported a negative association between serum TSH and the older resorption marker deoxypyridinoline but an absence of significant correlations to another resorption marker (ICTP) and the formation marker (PICP) [29].

Most of the studies above compared TSH values in and outside of the normal range. Our categorical analysis supports the notion that oversubstitution with LT4 causing total suppression of TSH causes accelerated bone loss as soon as TSH goes below the lower limit of normal, with the most pronounced increases seen in patients exhibiting fully suppressed TSH values. The impact between individuals, however, shows huge variations. Lesser reductions in TSH are associated with accelerated bone loss in some, but not all, patients affected. Our results also corroborate previous results from the studies cited above showing reduced bone loss in hypothyroidism.

Another factor affecting the dissimilarity in performance of separate bone markers may be differences in diurnal variation. The bone markers in this study were taken any time between 9am and 3pm in a nonfasting state. OC and BSAP show only minor diurnal variation amounting to 10–20% [30]. While PINP exhibits very small diurnal variations and minimal effects of feeding, CTX displays much larger daytime variations and is affected by feeding [31, 32]. Additionally, PINP is more stable at room temperature and has emerged as the most dynamic marker when it comes to assessing the effects of other pharmaceuticals on bone remodeling [33]. In this study, the possible confounding effects of daytime variation were offset by the grouping in the categorical analysis, but it would still add to variation. Moreover, in a recent study we showed that PINP can be assessed any time of day in a nonfasting state without significant bias, while CTX is still subject to variation, exhibiting a steady decrease from morning to afternoon [31]. This further strengthens the case for PINP as the optimal marker for assessment of bone turnover.

## 5. Conclusion

In conclusion, PINP emerges as the most sensitive marker of bone turnover in thyroid disorders. Thus, in cases where there is concern about possible adverse effects of thyroid hormone substitution therapy on bone, assessment of serum levels of PINP may be of value.

## Figures and Tables

**Table 1:** Demographics of the population studied.

Variable	*N*	Unit	Mean (SD)
Age	78	Years	60, 7 (11.5)
TSH	78	mU/L	1.05 (1.4)
FT4	78	*μ*mol/L	20.1 (15.8)
FT3	16	*μ*mol/L	6.8 (3.1)
25(OH)D	77	nmol/L	82.5 (33.5)
PTH	75	pmol/L	4.0 (2.0)
LS-BMD T-score	74	g/cm^2^	−0.87 (1.61)
TH-BMD T-score	73	g/cm^2^	−0.84 (1.13)
PINP	78	*μ*g/L	65.4 (33.2)
CTX	77	*μ*g/L	0.43 (0.30)
BSAP	75	*μ*g/L	12.3 (5.2)
OC	73	nmol/L	4.8 (3.3)

**Table 2:** Spearman's rho values for correlations of TSH, FT4, and FT3 on four bone markers: procollagen type 1 N-terminal propeptide (PINP); C-terminal cross-linking telopeptide of type 1 collagen (CTX); bone specific alkaline phosphatase (BSAP), and osteocalcin (OC).

BTM	TSH	FT4	FT3
PINP	−0.47 (*p* < 0.0001)	0.27 (*p*=0.018)	0.47 (*p*=0.066)
CTX	−0.38 (*p* < 0.001)	0.22 (*p*=0.056)	0.32 (*p*=0.248)
BSAP	−0.20 (*p*=0.088)	0.23 (*p*=0.046)	0.41 (*p*=0.142)
OC	−0.24 (*p*=0.037)	0.19 (*p*=0.103)	0.36 (*p*=0.270)

## Data Availability

The data used to support the findings of this study are available from the corresponding author upon reasonable request.

## Abbreviations

LS-BMD: Lumbar spine bone mineral density
TH-BMD: Total hip bone mineral density
PINP: Procollagen type 1 N-terminal propeptide
CTX: C-Terminal cross-linking telopeptide of type 1 collagen
BSAP: Bone specific alkaline phosphatase
OC: Osteocalcin.
